# Effect of a Co-Feed Liquid Whey-Integrated Diet on Crossbred Pigs’ Fecal Microbiota

**DOI:** 10.3390/ani13111750

**Published:** 2023-05-25

**Authors:** Anna Maria Sutera, Francesca Arfuso, Giuseppe Tardiolo, Valentina Riggio, Francesco Fazio, Riccardo Aiese Cigliano, Andreu Paytuví, Giuseppe Piccione, Alessandro Zumbo

**Affiliations:** 1 Department of Veterinary Sciences, University of Messina, Polo Universitario dell’Annunziata, Via Palatucci snc, 98168 Messina, Italy; asutera@unime.it (A.M.S.); francesca.arfuso@unime.it (F.A.); francesco.fazio@unime.it (F.F.); giuseppe.piccione@unime.it (G.P.); alessandro.zumbo@unime.it (A.Z.); 2 The Roslin Institute and Royal (Dick) School of Veterinary Studies, University of Edinburgh, Easter Bush Campus, Edinburgh EH25 9RG, UK; valentina.riggio@roslin.ed.ac.uk; 3 Sequentia Biotech SL, Carrer del Dr. Trueta 179, 08005 Barcelona, Spain; raiesecigliano@sequentiabiotech.com (R.A.C.); apaytuvi@sequentiabiotech.com (A.P.)

**Keywords:** crossbred pigs, liquid whey, next-generation sequencing, 16S rRNA, fecal microbiota, bacterial community, metagenomics

## Abstract

**Simple Summary:**

The use of low-cost by-products originating from agri- and dairy-chain production in animal nutrition represents an alternative strategy to feed and improve animal performance and reduce the environmental impact. In swine production, liquid-feeding products can positively contribute to the equilibrium of animal guts, thus supporting the reduction of feed medication. The liquid whey by-product of the cheese-making process is considered a palatable alternative feed and a promptly available source to provide functional ingredients without further costly transformation. In the last decade, increased knowledge of the gut microbiome has contributed to expanding our insights on host health, well-being, growth, and feed efficiency. Based on this perspective, this research investigated the fecal microbiota of crossbred pigs that underwent a co-feed liquid whey-integrated diet with a metagenomics approach.

**Abstract:**

This study assessed the potential effect of a co-feed liquid whey-integrated diet on the fecal microbiota of 14 crossbred pigs. The experimental design was as follows: seven pigs were in the control group, fed with a control feed, and seven were in the experimental group, fed with the same control feed supplemented daily with liquid whey. The collection of fecal samples was conducted on each animal before the dietary treatment (T0) and one (T1), and two (T2) months after the beginning of the co-feed integration. In addition, blood samples were collected from each pig at the same time points in order to evaluate the physiological parameters. Taxonomic analysis showed a bacterial community dominated by *Firmicutes*, *Bacteroidetes*, *Spirochaetes*, and *Proteobacteria* phyla that populated the crossbred pig feces. The diversity metrics suggested that the co-feed supplementation affected some alpha diversity indexes of the fecal microbiota. In addition, the differential abundance analysis at the genus level revealed significant differences for various genera, suggesting that the liquid whey supplementation potentially influenced a part of the bacterial community over time. Spearman’s correlations revealed that the differential abundant genera identified are positively or negatively correlated with the physiological parameters.

## 1. Introduction

Pigs are recognized worldwide as one of the most important livestock species, rep-resenting both a precious source of global meat production and a relevant animal model for studying the molecular background of several human diseases [1]. Because of the economic importance of pork meat, in the last few decades, many efforts have been directed towards genetic selection of this species as well as the improvement of management practices and nutrition [2,3,4]. Recent studies have shown that the mammals’ gut microbiota has numerous roles benefiting the host, such as the digestion and fermentation of carbohydrates, production of vitamins, maintenance of normal functions of the intestinal villi, regulation of the immune responses, and protection from pathogenic states [5,6]. The pig gut microbiota is a complex ecosystem showing dynamic composition and diversity, which can shift over time and along the entire gastrointestinal tract [7]. From birth to death, pig guts face changes according to their diet, habits, living environment, diseases, and related therapeutic treatments [8]. Pre-started diets potentially reduce the abrupt change in food encountered at weaning in suckling piglets [9,10]. As the pig is a species that is sensitive to stress, especially post-weaning, it is essential to optimize animal health and to use antibiotics in a more rational way, with an alternative to medication currently being represented by pre and probiotics [10].

The use of agro-industry by-products in livestock feeding has been widely explored in the last decades [11], aiming at low-cost support for production performances with positive effects on animal health and environmental sustainability [12]. Liquid whey (LW) is a by-product of the dairy industry with valuable nutritious properties that encourage its use as feed for livestock, representing a potential application of circular economy to the agri-food industry [13]. Although cheese-making processes can affect the chemical composition of LW, the proteins of this by-product are unique as they contain all the essential amino acids of a good quality protein [14]. It has been shown that its administration can affect body weight gain, feed efficiency, protein and fat digestibility, and mineral absorption and retention [15,16]. In light of this, this study explored the fecal microbiota of crossbred pigs that underwent a co-feed LW integrated diet. In addition, we evaluated the levels of inflammatory and immune markers such as serum haptoglobin, C-reactive protein, and white blood cell (WBC) count and correlated them with metagenomics data in order to explore how changes in the fecal microbiota could potentially affect animal health.

## 2. Materials and Methods

### 2.1. Animals Management and Experimental Design

The present study involved 14 crossbred pigs (Landrace × Large White) reared in an authorized farm located in Messina (Sicily, Italy) and housed in a barn, under controlled temperature (22 ± 2 °C) and relative humidity (60%), in individual pens with nipple waterers and stainless-steel feeders, and fed individually with free access to water. The 14 pigs were randomly divided in two groups, namely control (CTRL) and co-feed (LW) groups. The seven pigs in the CTRL group were fed for the duration of the trial with pelleted feed at 3% of their body weight (BW) per day. The seven pigs in the treated group received the same pellet feed, supplemented with 1.5 L of LW per day/pig. The nutritional composition of both pellet feed and LW, as well as the amino acids levels of the diet are reported in Table S1. The dairy by-product was administered daily as co-feed using a wet feeder. The pigs in both groups consumed all of the feed provided per day. The pigs’ growth performance was determined by the average daily gain (ADG) and food conversion rate (FCR). Every 30 days, BW was measured in the morning before being fed, at the same time points when feces samples were collected (see Section 2.2). Groups were homogeneous for sex (female), BW (average initial BW of 20 ± 1.5 kg), age (60 ± 2 days), and management. The animals were healthy and no exposure to antibiotics was recorded before the beginning of the trial. The study lasted over 60 days after an initial adaptation to the diet of 15 days.

### 2.2. Blood and Fecal Sampling and Next Generation Sequencing

Individual blood samples were collected by jugular venipuncture into both EDTA-vacutainer tubes and tubes with a cloth activator (Terumo Corporation, Tokyo, Japan), so as to assess WBC count and the concentration of haptoglobin and C-reactive protein fractions, respectively, as described by D’Alessandro et al. [17]. A total of 42 fecal samples were collected from the rectal ampoule of the 14 pigs involved in the trial, before the administration of the treatment (T0) and one (T1), and two (T2) months after (i.e., three time points per pig), using sterile plastic tubes. Once transported to the laboratory, for each sample, an aliquot of 400 mg was stored in OMNIgene^®^•GUT tubes (Voden Medical Instruments, Meda, Italy). Microbial genomic DNA extraction and 16S-amplicon sequencing of the V3-V4 hypervariable region were performed at Eurofins Genomics (Konstanz, Germany) using Illumina’s MiSeq v3 platform in 2 × 300 bp paired-end mode (San Diego, CA, USA). 

### 2.3. Bioinformatics and Statistical Analysis

The quality of Illumina’s raw reads was assessed using FastQC [18]. Trimmomatic software v0.39 [19] was subsequently used to remove Illumina adapters, as well as all the low-quality reads with a Phred score ≤ 20, filtering for a minimum read length of 50 and trimming low-quality 3′ ends of reads, as previously described by Tardiolo et al. [20]. The GAIA pipeline [21] was used for the bioinformatics analysis. The taxonomic assignments performed by GAIA were obtained using a mapping-based approach against a custom-made database from NCBI, followed by a Lowest Common Ancestor algorithm according to Paytuví et al. [21]. Subsequently, the generated Operational Taxonomic Units (OTUs) table was uploaded in plain format to the MicrobiomeAnalyst tool [22,23] together with a taxonomy file for the corresponding OTUs and the information associated to each sample (i.e., metadata) for statistical analysis and visualization. Features across samples at different taxonomic levels were filtered based on their abundance level (i.e., minimum count 2) and sample prevalence (i.e., 20% prevalence in all of the samples). The t-test ANOVA was used to estimate alpha diversity indexes based on Observed species, Chao1, Shannon, and Simpson. Beta diversity community was determined via the Bray-Curtis index using a PERMANOVA statistics and was visualized with principal coordinate analysis (PCoA) plot. Differential abundance analysis was performed in RStudio [24] using the DESeq2 package [25] to identify significant differences by correlating the co-feed integration over time. An adjusted *p*-value ≤ 0.05 was considered statistically significant for the aforementioned statistical methods. Regarding the physiological parameters, recorded data were statistically analyzed and expressed in terms of mean values of the three replications of each variable and standard errors. Two-way analysis of variance was conducted for hypothesis testing at 1% level of significance. Post hoc Tukey’s HSD test was performed for a mean comparison between the groups across time points in relation to the dependent variables. Spearman’s correlations were calculated to evaluate the association between the physiological parameters across the two feeding groups. All statistical analyses for the physiological parameters were performed in RStudio [24]. 

## 3. Results

### 3.1. Quality Control and Taxonomic Profile of the Bacterial Communiy

**Figure 1:**
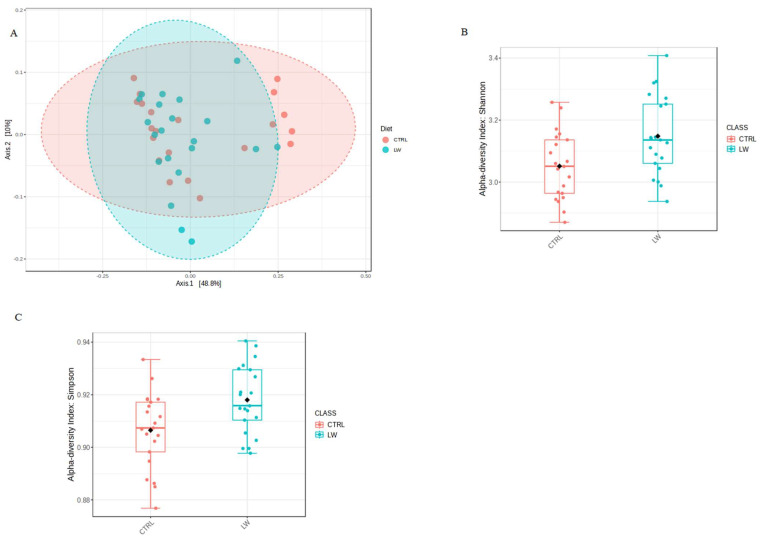
(**A**) PCoA plot based on Bray-Curtis distances of the fecal microbiota community for the co-fed pigs (LW) and the CTRL group. (**B**) Shannon index (*p* = 0.01) and (**C**) Simpson index (*p* = 0.009).

After Illumina sequencing, the total number of filtered sequences obtained was 1,609,200 with a median sequencing coverage of 38,314 sequences. The minimal and maximal coverages were 24,876 and 53,950 reads. Figure S1 reports the rarefaction curve of all samples, showing that an adequate sequencing depth was obtained. The V3–V4 hypervariable region of the 16S rRNA gene was sequenced from 42 fecal samples of crossbred pigs collected at three time points. Sequences processing using GAIA pipeline [21] revealed 938 OTUs at the genus level among all samples. The PCoA plot based on the Bray-Curtis distance of the fecal bacteria community showed that the samples of the two feeding groups did not cluster separately (Figure 1A), and the distances were not significantly different with an overall *p*-value = 0.056 (R^2^ = 0.05). Regarding the alpha diversity indexes, the Shannon and Simpson indices were significantly higher in the co-feed animals (*p* = 0.01 and 0.009, respectively) compared to the control ones (Figure 1B,C).

**Figure 2:**
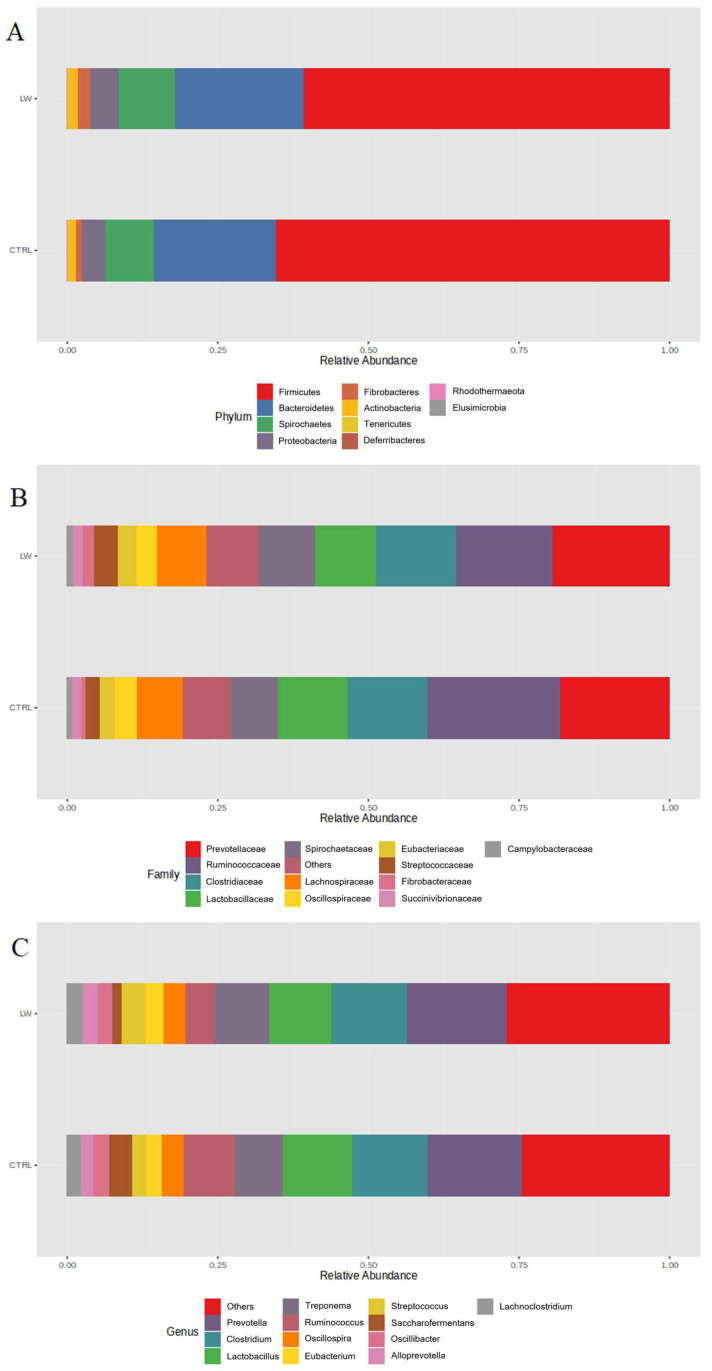
Relative abundances of phyla (**A**), families (**B**), and genera (**C**) observed in the co-feed (LW) group compared with those of the control group (CTRL). Only the most represented taxa are reported.

At the phylum level, the bacterial community of the pigs in the control group was dominated by *Firmicutes* (65%), followed by *Bacteroidetes* (22%), *Spirochaetes* (8%), and *Proteobacteria* (4%). However, pigs receiving the co-feed supplementation were dominated by the same phyla (60%, 20%, 9%, and 5%, respectively) (Figure 2A).

At the family level, *Prevotellaceae* (18%)*, Ruminococcaceae* (22%),* Clostridiaceae* (13%)*, Lactobacillaceae* (11%), and *Spirochaetaceae* (8%) were the most abundant in the control group (Figure 2B). The same families dominated in the treated pigs (20%, 16%, 13%, 10%, and 9%, respectively). Similarly, the most represented genera detected in the CTRL group were also the most represented in the LW group (Figure 2C); namely, *Prevotella* (16% and 15%, for CTRL and LW, respectively), *Clostridium* (12% for both), *Lactobacillus* (11% and 10% for CTRL and LW, respectively), and *Treponema* (8% and 9% for CTRL and LW, respectively).

### 3.2. Differential Abundance Analysis of Bacterial Genera

Differential abundance analysis was performed using the DEseq2 package in RStudio to evaluate potential changes at the genus level. As a result, 42 genera were encountered significantly different, correlating the co-feed integration over time (Table S2). Among them, those mainly modulated are reported in Table 1, referring to the co-feed integration over time (T2 vs. T0). The genera most positively modulated over time in the co-feed group were *Bifidobacterium*, *Parasutterella*, *Oxalobacter*, *Lactobacillus*, *Cellulosilyticum*, *Ruminococcus*, *Petrimonas*, and *Rubrivirga,* and the most negatively were *Mogibacterium*, *Faecalibacterium*, *Collinsella*, *Oribacterium*, *Mediterranea*, *Gemmiger*, *Coprococcus*, *Sutterella*, *Slackia*, *Butyricicoccus*, and *Corynebacterium*.

### 3.3. Growth Performance and Physiological Parameters

**Figure 3:**
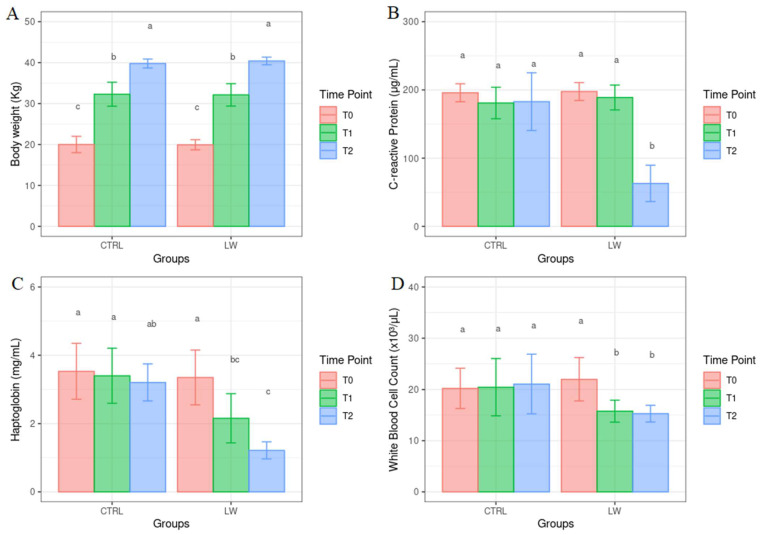
Mean values ± standard error for body weight (**A**), C-reactive protein (**B**), haptoglobin (**C**) and white blood cell count (**D**) from control and co-feed pigs before (T0), as well as one (T1) and two (T2) months after the co-feed liquid whey supplementation (a, b and c show the statistically significant differences among variables).

All results correlated to the physiological parameters are reported as the mean ± standard error of the mean. BW showed a significant increasing trend (Figure 3) both in the CTRL and LW group (*p* < 0.01), with no significant effect of the diet observed (*p* > 0.05). In the control group, the ADG was 330 g/head per day and 340 g/head per day in the co-feed animals. The average daily feed intake was 897 g in the control and 906 g in the treated animals. FCR was 2.71 kg/kg and 2.66 kg/kg in the control and co-feed pigs, respectively. The co-feed supplementation had an effect on the serum concentration values of haptoglobin and C-reactive protein (*p* < 0.0001) and WBC count (*p* < 0.02) (Figure 3). Moreover, a significant decreasing trend (*p* < 0.01) for haptoglobin, C-reactive protein, and WBC count was observed in the co-feed group from T0 to T2 (Figure 3). On the other hand, in the control group, no significant variation over time (*p* > 0.05) was observed.

### 3.4. Correlation between Differential Abundant Genera and Physiological Parameters

**Figure 4:**
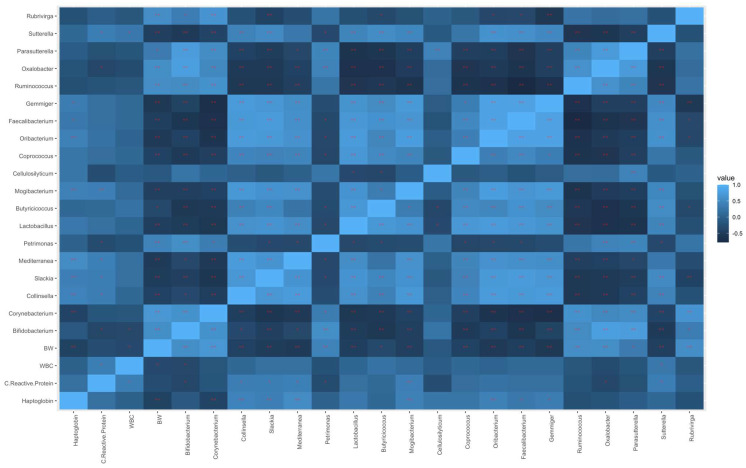
Heatmap representing Spearman’s correlations between the most modulated genera and the physiological parameters (* *p* < 0.05 and ** *p* < 0.01).

Spearman’s correlations were calculated between the physiological parameters and the most modulated genera identified in the differential abundance analysis based on the co-feed integration over time, as reported in Figure 4.

Positive correlations were found between BW and *Bifidobacterium* (r = 0.56; *p* < 0.01), *Corynebacterium* (r = 0.67; *p* < 0.01), *Petrimonas* (r = 0.44; *p* < 0.01), *Ruminoccoccus* (r = 0.55; *p* < 0.01), *Oxalobacter* (r = 0.55; *p* < 0.01), *Parasutterella* (r = 0.33; *p* < 0.05), and *Rubrivirga* (r = 0.55; *p* < 0.01). On the other hand, BW was negatively correlated to *Collinsella* (r = −0.44; *p* < 0.01), *Slakia* (r = −0.52; *p* < 0.01), *Mediterranea* (r = −0.53; *p* < 0.01), *Lactobacillus* (r = −0.49; *p* < 0.01), *Butyricicoccus* (r = −0.35; *p* < 0.05), *Mogibacterium* (r = −0.46; *p* < 0.01), *Coprococcus* (r = −0.42; *p* < 0.01), *Oribacterium* (r = −0.56; *p* < 0.01), *Faecalibacterium* (r = −0.48; *p* < 0.01), *Gemmiger* (r = −0.59; *p* < 0.01), and *Sutterella* (r = −0.46; *p* < 0.01). Regarding the C-reactive protein, positive correlations were observed with *Collinsella* (r = 0.37; *p* < 0.05), *Slackia* (r = 0.38; *p* = *p* < 0.05), *Mediterranea* (r = 0.39; *p* < 0.05), *Mogibacterium* (r = 0.40; *p* < 0.01), and *Sutterella* (r = 0.38; *p* = 0.05), whereas negative correlations were found with *Bifidobacterium* (r = −0.37; *p* < 0.05), *Petrimonas* (r = −0.31; *p* < 0.05), and *Oxalobacter* (r = −0.37; *p* < 0.05). *Collinsella* (r = 0.43; *p* < 0.01), *Slackia* (r = 0.40; *p* < 0.01), *Mediterranea* (r = 0.52; *p* < 0.01), *Mogibacterium* (r = 0.41; *p* < 0.01), *Oribacterium* (r = 0.40; *p* < 0.01), *Faecalibacterium* (r = 0.31; *p* < 0.05), and *Gemmiger* (r = 0.35; *p* < 0.05) genera were positively correlated with haptoglobin, whereas *Corynebacterium* (r = −0.41; *p* < 0.01) was negatively correlated. The WBC count showed a moderate positive correlation with the *Sutterella* (r = 0.31; *p* < 0.05) genus, and a moderate negative correlation with *Bifidobacterium* (r = −0.35; *p* < 0.05).

## 4. Discussion

The continuously increasing of the global population requires raising overall food production to meet the global food demand. Unfortunately, it has been estimated that approximately one-third of the food produced worldwide for human consumption is wasted, representing a significant loss of the resources spent making, processing and transporting food. Therefore, the use of agro-industry by-products in livestock feeding is considered a potential strategy to reduce food waste, as well as having an influence on growth performance, immune function, and product quality. However, it is quite difficult to generalize on the efficiency of these by-products, due to their different origin and the complexity of their chemical composition; it is also essential to make sure they do not have negative effects on the animals and their performances.

A progressive increase in BW was observed in both the control and co-feed pigs. Although this is not a surprising result per se, as BW was recorded at different time points (i.e., it is expected that animals grow in weight with age), it is suggesting that LW supplementation did not negatively affect the weight of pigs in the fattening stage of their growth, thus confirming their good health status. On the other hand, the co-feed diet influenced the other physiological parameters investigated, with WBC count, haptoglobin, and C-reactive protein being lower in co-feed pigs.

The analysis of the fecal microbiota profile revealed 938 OTUs identified at the genus level among all of the samples. From a taxonomic point of view, our results on phyla, families, and genera composition (Figure 2) are generally comparable with those reported in other studies [20,26,27,28]. The alpha diversity analysis of the bacterial community suggested that the co-feed treatment affected some indices, with the Shannon and Simpson values being statistically significant between the feeding conditions (Figure 1B,C). However, PCoA plot based on the Bray-Curtis distance did not reveal remarkable differences (Figure 1A).

As the mucosa and other parts of the intestine are not easily accessible, it is common practice to carry out the studies on gut microbiota using feces, based on the knowledge that feces contain mainly the luminal microbiota of the distal colon, whereas the mucosal-associated microbiota is likely to have a more substantial physiological influence on the host [29]. In our study, both the control and co-feed group were dominated by *Firmicutes* (65%) followed by *Bacteroidetes* (22%), *Spirochaetes* (8%), and *Proteobacteria* (4%) phyla. Adhikari et al. [30] reported *Proteobacteria* being significantly higher in the mucosa as compared with the lumen in nursery pigs, as also shown by Burrough et al. [31] and Mu et al. [32] in both healthy and diseased adult and nursery pigs, respectively. The same authors (i.e., Adhikari et al. [30]) also reported *Bacteroidetes* and *Firmicutes* being highly associated with the mucosa and lumen, respectively. However, we did not have data to assess the association between the fecal and intestinal microbiota (either mucosal-associated or luminal) in the animals used in our study.

When considering the samples at different time points, the differential abundance analysis at genus level correlating the co-feed integration over time revealed that several genera were positively or negatively modulated (Table 1). Among those positively modulated, we identified several species belonging to beneficial genera such as *Lactobacillus*, *Bifidobacterium* and *Oxalobacter* that are exploited as probiotics and in feed additives production to prevent diarrhea, improve growth, regulating immune function, and counteracting potential pathogenic states [33,34,35,36]. It could be hypothesized that the higher presence of *Lactobacillus* species in the LW group is due to the nature of the by-product used (i.e., from cheese-making industry). In a study to evaluate the effect of *L. acidophilus* supplementation in weaning pigs, Lan et al. [37] have shown that pigs fed this supplementation diet increased ADG and gain:feed ratio. Moreover, *L. acidophilus* supplementation led to an increase in dry matter digestibility and shift microbiota by increasing fecal *Lactobacillus*, while decreasing *E. coli* counts, as well as a decrease in serum blood urea nitrogen concentration and fecal noxious gas emission [37].

Furthermore, Spearman’s correlations showed that some differentially abundant genera over time were positively or negatively correlated with the physiological parameters considered (Figure 4). Interestingly, some of the genera that were positively correlated with BW had a negative correlation with the other parameters (i.e., C-reactive protein, haptoglobin, and WBC count), such as *Bifidobacterium*, *Corynebacterium*, *Petrimonas*, and *Oxalobacter*; and those with a negative correlation with BW had a positive correlation with the other parameters, such as *Collinsella*, *Slakia*, *Mediterranea*, *Mogibacterium*, *Oribacterium*, *Faecalibaterium*, *Gemmiger,* and *Sutterella*. However, this was perhaps to be expected as BW is negatively correlated with C-reactive protein, haptoglobin, and WBC count. Some of these genera have already been reported in the literature as playing an important role in the gut microbiota of several species. The presence of the *Petrimonas* genus has been reported in the bacterial community of pigs naturally resistant to African swine fever [38]. In the human gastrointestinal tract and in several animal models, the genus *Parasutterella* has been characterized as a member of the healthy fecal core microbiome [39,40]. In agreement with our results, *Corynebacterium*, a genera potentially associated with pathogenic states, was previously reported to be positively correlated with BW and feed efficiency in pigs [41]. A negative correlation between BW and *Collinsella* has been also reported by Miragoli et al. [28]. Several studies have shown that *Collinsella* achieves permanent colonization of the gut mucosa in both pigs and humans via the utilization of mucins [42,43], thus indicating a direct interaction between these microorganisms and the intestinal tissue of the host [28]. Similarly, Miragoli et al. [28] found a negative correlation, although not significant, between BW and *Mogibacterium*, which has previously been observed to increase in the mucosa-associated microbiota of colon cancer patients [44]. Interestingly, this genus, along with *Collinsella*, decreased in the feces of newborn pigs that received a beneficial prebiotic formulation [45].

Among the genera negatively correlated with BW, *Faecalibacterium* and *Gemmiger* have been reported, in a large-scale analysis using population-based studies in humans, as strongly associated with diet [46]. Surprisingly, we identified the *Mediterranea* genus that has been previously reported in humans as a new candidate genus belonging to the *Bacteroidaceae* family [47,48]. Finally, the *Sutterella* genus was associated with gastrointestinal diseases, inducing substantial inflammation and as a dominant genus in diarrheal pigs [49].

Although not significantly correlated with any of the physiological parameters, we found *Cellulosilyticum* as one of the most positively modulated genera over time in the co-feed group. This genus is known to be a symbiont for the degradation of dietary fiber during the late growth phase of pigs, and could thus be helpful in promoting digestive processes in the gut [41]. Interestingly, similar results were also observed in an autochthonous pig breeds co-fed using LW [20]. The WBC count showed a moderate positive correlation with *Sutterella* and a negative correlation with the *Bifidobacterium* genera, suggesting a role of these genera in the immune status of the host. Moreover, as high levels of C-reactive protein and haptoglobin are associated with inflammatory states, it can be hypothesized that the *Bifidobacterium*, *Petrimonas*, *Oxalobacter*, *Collinsella*, *Slackia*, *Mediterranea*, *Mogibacterium*, *Oribacterium*, *Faecalibacterium*, *Corynebacterium,* and *Gemmiger* genera play a role in acute phase response. C-reactive protein and haptoglobin are indeed positive acute proteins as their serum levels increase during the acute phase response or inflammation, as previously shown in other studies [50,51].

Finally, our results suggest that the use of LW as a supplemental feed could affect pigs’ fecal microbiota by positively modulating the beneficial bacteria and potentially reducing the harmful taxa, as similarly reported by Tardiolo et al. [20]. Furthermore, the correlation analysis confirmed the relationship between bacteria and leukocytes and/or acute-phase proteins.

## 5. Conclusions

Our results have shown that the use of liquid whey as a supplemental feed did not have an effect on pigs’ growth performance for the duration of the study. However, as the Mediterranean basin economy is generally agriculture-based, with the dairy industry representing a good proportion, we believe that the use of low-cost dairy by-products (such as liquid whey) to feed the animals can have a significant economic impact on the territory, especially for small to medium-sized farms. We have also shown that, although co-feed supplementation with the dairy by-products likely affected a part of the microbial diversity, its supplementation over time affected the abundance of some beneficial genera of the fecal microbiota. Our findings indicate that a low-cost supplemented diet using a dairy by-product as co-feed, such as liquid whey, might be potentially employed in swine production in order to improve animal health. In addition, this alternative strategy represents an added value of supporting the reduction of environmental impact. Further studies focusing on functional study at a species level could better elucidate the complex interaction between the bacterial community and host metabolism.

## Figures and Tables

**Table 1:** Differential abundance analysis reporting significant variation at the genus level over time, sorted according to the adjusted *p*-value.

Genus	Log2 Fold Change *	*p*-Value	Adjusted *p*-Value
*Bifidobacterium*	2.7558	3.97 × 10^−15^	7.93 × 10^−13^
*Parasutterella*	3.0901	3.12 × 10^−13^	3.12 × 10^−11^
*Mogibacterium*	−2.8128	3.07 × 10^−11^	2.05 × 10^−9^
*Oxalobacter*	5.8482	1.28 × 10^−7^	6.39 × 10^−6^
*Faecalibacterium*	−1.7174	2.49 × 10^−7^	9.81 × 10^−6^
*Lactobacillus*	1.1807	2.94 × 10^−7^	9.81 × 10^−6^
*Collinsella*	−4.6674	5.23 × 10^−6^	1.19 × 10^−4^
*Oribacterium*	−2.1668	5.40 × 10^−6^	1.19 × 10^−4^
*Mediterranea*	−3.8855	1.92 × 10^−5^	3.19 × 10^−4^
*Gemmiger*	−1.4709	3.03 × 10^−5^	4.66 × 10^−4^
*Cellulosilyticum*	2.7283	5.98 × 10^−5^	8.54 × 10^−4^
*Coprococcus*	−1.9272	6.70 × 10^−5^	8.93 × 10^−4^
*Sutterella*	−1.3979	1.25 × 10^−4^	1.54 × 10^−3^
*Slackia*	−2.9637	1.32 × 10^−4^	1.54 × 10^−3^
*Ruminococcus*	−1.3224	3.81 × 10^−4^	3.99 × 10^−3^
*Butyricicoccus*	−1.1101	4.33 × 10^−4^	4.12 × 10^−3^
*Petrimonas*	3.8546	9.72 × 10^−4^	8.45 × 10^−3^
*Corynebacterium*	−2.1777	1.93 × 10^−3^	1.43 × 10^−2^
*Rubrivirga*	2.8509	9.76 × 10^−3^	4.54 × 10^−2^

* Changes in genus-level abundance refer to co-feed integration over time (T2 vs. T0).

## Data Availability

Supporting data of this study are available from the corresponding authors upon reasonable request. Sequencing data have been deposited into the Sequence Read Archive database under the study accession number SUB12998317 associated with the BioProject ID PRJNA951774.

## Supplementary Materials

The following supporting information can be downloaded at: https://www.mdpi.com/article/10.3390/ani13111750/s1. Table S1: Ingredients and nutritional composition of the diet. Figure S1: Rarefaction curve graph. Table S2: Differential abundance analysis reporting the significant variation at the genus.
