# Supplementary material for: Effect of a Co-Feed Liquid Whey-Integrated Diet on Crossbred Pigs’ Fecal Microbiota
Source: Animals (Basel). 2023 May 25;13(11):1750. doi: 10.3390/ani13111750 (PMC10252047; doi:10.3390/ani13111750)
Supplement: Supplementary file 1 [file animals-13-01750-s001.zip › Table_S1_Ingredients_and_nutritional_composition_of_the_diet.pdf]

Table S1. Ingredients and nutritional composition of the diet.

Ingredients	g/kg of dry matter
Corn	550
Broad bean	125
Peas bean	110
Sunflower meal (38% crude protein)	80
Wheat middling	70
Carob	30
Sugar cane molasses	13
**Analytical components**	% on a wet basis
Crude protein	17.4
Crude fat	5.7
Crude fiber	4.5
Ash	5.3
Calcium	0.6
Phosphorus	0.5
Sodium	0.2
Lysine	1.2
Methionine + Cystine	0.57
Tryptophan	0.17
Threonine	0.62
**Additive components**	
Vitamin B1	1.0 mg
Vitamin B2	3.0 mg
Vitamin B6	1.5 mg
Vitamin B12	0.015 mg
Vitamin D3	(1.000 UI)
Vitamin E	20 mg
Vitamin K3	1.0 mg
Niacin	15.0 mg
Calcium-D	10.3 mg
Choline	200 mg
Cu	14.0 mg
Fe	89.8 mg
I	0.50 mg
Mn	39.9 mg
Se	0.15 mg
Zn	99.6 mg
Biotin	0.10 mg
**Liquid whey chemical composition**	
Fat	1.3 %
Protein	2.7 %
Lactose	4.5 %
Solids non-fat	7.5 %
Ca	47 mg/L
Mg	9 mg/L
Cl	38 mg/L
K	192 mg/L
Na	92 mg/L
NaCl	60 mg/L
pH	4.9
Density	27.85

Data reported were provided by the commercial farm.
