# Supplementary material for: Effect of a Co-Feed Liquid Whey-Integrated Diet on Crossbred Pigs’ Fecal Microbiota
Source: Animals (Basel). 2023 May 25;13(11):1750. doi: 10.3390/ani13111750 (PMC10252047; doi:10.3390/ani13111750)
Supplement: Supplementary file 1 [file animals-13-01750-s001.zip › Table_S2_Differential_abundance_analysis_reporting_the_significant_variation_at_genus_level.pdf]

Table S2. Differential abundance analysis reporting the significant variation at genus level.

Genus	Log2 Fold Change*	p-value	adjusted p-value
Bifidobacterium	2,7558	3,97 x 10^{-15}	7,93 x 10^{-13}
Parasutterella	3,0901	3,12 x 10^{-13}	3,12 x 10^{-11}
Mogibacterium	-2,8128	3,07 x 10^{-11}	2,05 x 10^{-09}
Oxalobacter	5,8482	1,28 x 10^{-07}	6,39 x 10^{-06}
Faecalibacterium	-1,7174	2,49 x 10^{-07}	9,81 x 10^{-06}
Lactobacillus	1,1807	2,94 x 10^{-07}	9,81 x 10^{-06}
Campylobacter	1,372	1,22 x 10^{-06}	3,49 x 10^{-05}
Collinsella	-4,6674	5,23 x 10^{-06}	1.19 x 10^{-04}
Oribacterium	-2,1668	5,40 x 10^{-06}	1.19 x 10^{-04}
Lachnobacterium	-1,6558	1,57 x 10^{-05}	3.13 x 10^{-04}
Fournierella	-1,9893	1,73 x 10^{-05}	3.14 x 10^{-04}
Mediterranea	-3,8855	1,92 x 10^{-05}	3.19 x 10^{-04}
Gemmiger	-1,4709	3,03 x 10^{-05}	4.66 x 10^{-04}
Cellulosilyticum	2,7283	5,98 x 10^{-05}	8.54 x 10^{-04}
Coprococcus	-1,9272	6,70 x 10^{-05}	8.93 x 10^{-04}
Sutterella	-1,3979	1.25 x 10^{-04}	1.54 x 10^{-03}
Slackia	-2,9637	1.32 x 10^{-04}	1.54 x 10^{-03}
Alistipes	1,0593	1.39 x 10^{-04}	1.54 x 10^{-03}
Ruminococcus	1,3224	3.81 x 10^{-04}	3.99 x 10^{-03}
Lachnoclostridium	0,60017	3.99 x 10^{-04}	3.99 x 10^{-03}
Butyricicoccus	-1,1101	4.33 x 10^{-04}	4.12 x 10^{-03}
Fibrobacter	1,1034	9.28 x 10^{-04}	8.44 x 10^{-03}
Petrimonas	3,8546	9.72 x 10^{-04}	8.45 x 10^{-03}
Ruthenibacterium	-1,535	1.62 x 10^{-03}	1.34 x 10^{-02}
Parabacteroides	0,77954	1.72 x 10^{-03}	1.34 x 10^{-02}
Solitalea	1,5431	1.74 x 10^{-03}	1.34 x 10^{-02}
Corynebacterium	-2,1777	1.93 x 10^{-03}	1.43 x 10^{-02}
Lachnospira	-1,3854	2.26 x 10^{-03}	1.58 x 10^{-02}
Ruminiclostridium	0,6677	2.30 x 10^{-03}	1.58 x 10^{-02}
Phascolarctobacterium	1,2654	3.38 x 10^{-03}	2.23 x 10^{-02}
Anaerotruncus	0,84996	3.57 x 10^{-03}	2.23 x 10^{-02}
Enterorhabdus	-1,8791	3.58 x 10^{-03}	2.23 x 10^{-02}
Angelakisella	0,78807	3.72 x 10^{-03}	2.25 x 10^{-02}
Paraeggerthella	-1,9912	3.96 x 10^{-03}	2.26 x 10^{-02}
Oscillospira	0,55307	3.99 x 10^{-03}	2.26 x 10^{-02}
Peptococcus	-1,0756	4.08 x 10^{-03}	2.26 x 10^{-02}
Dorea	-1,1646	5.70 x 10^{-03}	3.08 x 10^{-02}
Taibaiella	1,1028	7.73 x 10^{-03}	4.02 x 10^{-02}
Anaerobium	1,579	7.84 x 10^{-03}	4.02 x 10^{-02}
Blautia	-0,79387	8.87 x 10^{-03}	4.36 x 10^{-02}
Filobacterium	2,8164	8.95 x 10^{-03}	4.36 x 10^{-02}
Bacteroides	1,0571	9.57 x 10^{-03}	4.54 x 10^{-02}
Rubrivirga	2,8509	9.76 x 10^{-03}	4.54 x 10^{-02}

* changes in genus-level abundance refer to co-feed integration over time (T2 *vs* T0).
