# Supplementary figures and images for: Effect of a Co-Feed Liquid Whey-Integrated Diet on Crossbred Pigs’ Fecal Microbiota
Source: Animals (Basel). 2023 May 25;13(11):1750. doi: 10.3390/ani13111750 (PMC10252047; doi:10.3390/ani13111750)

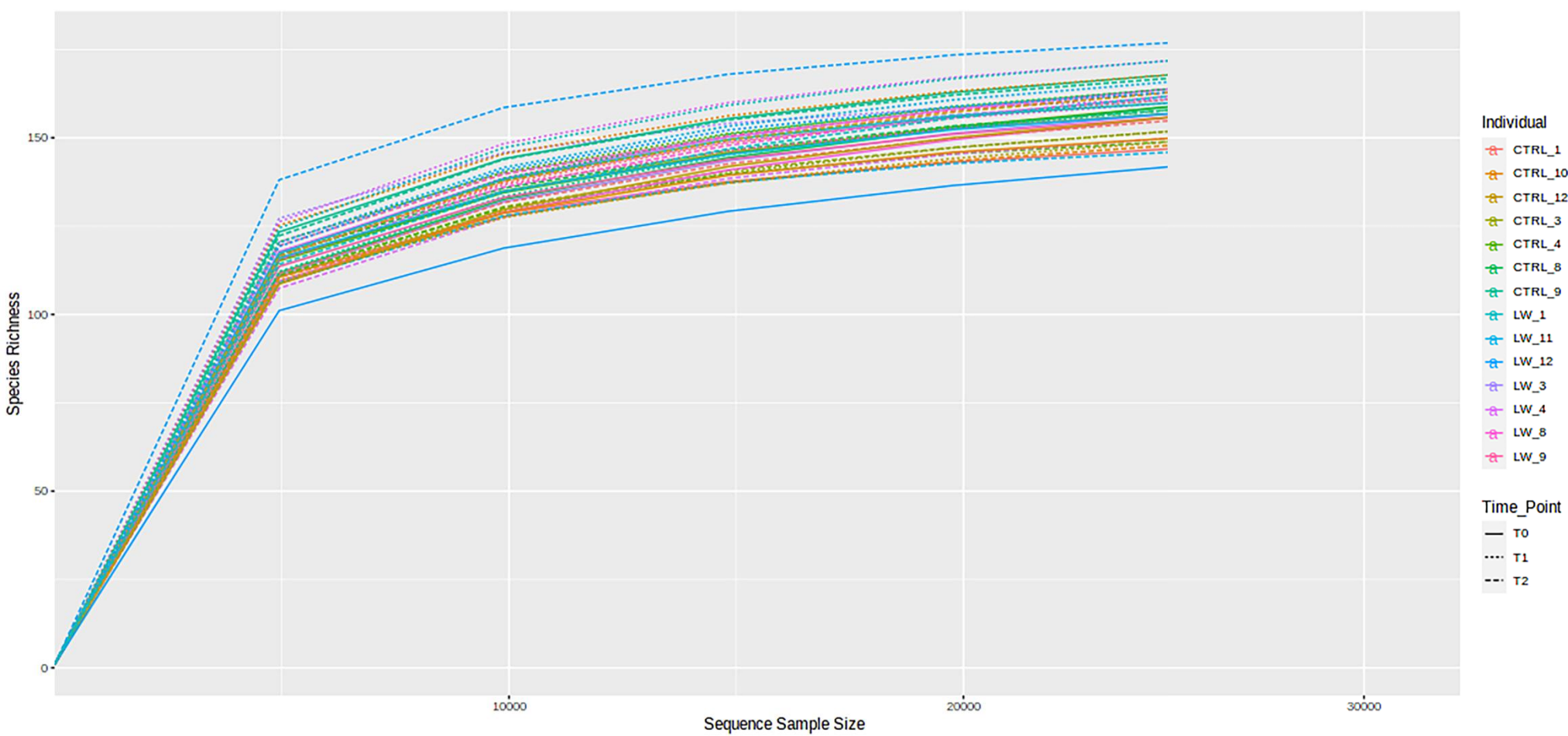

Supplement: Supplementary file 1 [file animals-13-01750-s001.zip › Figure_S1_Rarefaction_curve_graph.pdf]
